# Salivary Microbiota Is Significantly Less Diverse in Patients with Chronic Spontaneous Urticaria Compared to Healthy Controls: Preliminary Results

**DOI:** 10.3390/life11121329

**Published:** 2021-12-01

**Authors:** Diana Ćesić, Liborija Lugović-Mihić, Iva Ferček, Ana Gverić Grginić, Marko Jelić, Iva Bešlić, Arjana Tambić Andrašević

**Affiliations:** 1 Department of Dermatology and Venereology, Sestre Milosrdnice University Hospital Centre, 10 000 Zagreb, Croatia; liborija@sfzg.hr (L.L.-M.); ivaaabukvic@gmail.com (I.B.); 2 School of Dental Medicine, University of Zagreb, 10 000 Zagreb, Croatia; arjana.tambic@bfm.hr; 3 Department of Ophthalmology, Sestre Milosrdnice University Hospital Centre, 10 000 Zagreb, Croatia; iva.fercek@gmail.com; 4 Department of Clinical Microbiology, Sestre Milosrdnice University Hospital Centre, 10 000 Zagreb, Croatia; ana.gveric.grginic@kbcsm.hr; 5 Department of Clinical Microbiology, University Hospital for Infectious Diseases, 10 000 Zagreb, Croatia; mjelic@bfm.hr

**Keywords:** salivary microbiota, urticaria, chronic spontaneous urticaria, 16S rRNA gene, biodiversity, oral-gut axis, saliva

## Abstract

Background: Because of the important role in regulating the immune system, increasing evidence suggests a possible implication of gut microbiota in Chronic spontaneous urticaria (CSU). Although the oral cavity is the first site of contact between microbiota and the immune system, the association between salivary microbiota and CSU has not yet been reported. Objective: This case-control study aimed to compare differences in salivary microbiota between CSU patients and healthy controls (HC). Twenty-three participants—13 patients with CSU and 10 HC were enrolled; salivary microbiota was determined by molecular approach targeting 16S ribosomal RNA. Terminal restriction fragment length polymorphism (T-RFLP) analysis was performed. Results: Alpha diversity of salivary microbiota in CSU patients was significantly reduced compared to HC, resulting in alteration of the community composition. Species richness determined via the Shannon index was significantly reduced in the CSU group. Conclusion: Dysbiosis of salivary microbiota may contribute to a dysregulated immune system in the development of CSU. To our knowledge, this was the first study that reported an alteration in salivary microbiota composition in CSU patients.

## 1. Introduction

Chronic urticaria (CU) is defined as a continuous or intermittent occurrence of wheals, angioedema, or both for more than 6 weeks [1]. The prevalence of CU is increasing worldwide, with an overall point prevalence of 0.7%, ranging from 0.1% to 1.5% in adults [2,3]. Children population is affected in a similar proportion, with prevalence ranging from 0.1% to 3% [4]. Women are affected nearly twice as often as men, with the peak age between 20 and 40 years [5].

Based on the relevance of triggering factors, CU is classified into two categories–chronic spontaneous urticaria (CSU) and chronic inducible urticaria (CInd) [1]. Approximately 80% of CU cases are CSU with no identified triggering stimuli or specific allergens [6]. Debilitating symptoms and often unsuccessful treatment affect patients’ performance at work and school and significantly impair quality of life [2]. The course of the disease can be self-limiting, although, in 10–25% of patients, it lasts longer than 5 years [7].

Although many advances have been made in identifying etiopathogenetic mechanisms of CSU, the etiology and pathogenesis are complex and remain largely unclear. The abnormal activation of mast cells and basophils is the key process in CSU development [8]. Autoimmunity type I (IgE autoantibodies to IL-24, thyroperoxidase, double-stranded DNA, and other autoallergens) or type IIb (IgG antibodies to the patient’s own IgE or its high-affinity receptor-FcεRI) are involved in a large proportion of CSU cases [8], but also other factors have been elucidated: infections, pseudoallergic reactions, coagulation, stress, vitamin D [9]. Several recent studies have been identified altered gut microbiota as a possible underlying cause of CSU [6,10,11,12,13,14].

The oral cavity is a significant gateway to the human body. It comprises different microbiological habitats (saliva, buccal mucosa, palatal mucosa, tongue dorsum, dental surfaces) that form the salivary or oral microbiota [15]. The microbial community of the oral cavity is the second most complex of the human body after the gut microbiome. To date, over 700 oral bacterial species have been identified in the human oral microbiome database (HOMD) [16]. The microorganisms residing in the oral cavity, and their inter-relationships, are essential components in changing the balance between health and disease. Microorganisms colonizing one area of the oral cavity have a significant probability of spreading on contiguous epithelial surfaces to neighboring sites [15]. Saliva can be rapidly collected from subjects and immediately preserved, allowing accurate profiling of taxonomic changes in response to different treatments or disease states. It is also convenient for research since the use of saliva samples is inexpensive and non-invasive. Therefore, salivary microbiota has become one of the most studied microbiota to date. Many recent studies have reported alterations in salivary microbiota for various diseases, including obesity [17], rheumatoid arthritis [18], type 2 diabetes mellitus [19], atherosclerosis [20], etc. Also, it is important to mention increasing evidence suggesting that some oral bacteria may play a physiological role in digesting gluten in patients with celiac disease; enzymes produced by oral bacteria are capable to modify immunologically important gliadin peptides and the way they present to the gut immune system [21].

Even though the oral cavity is the first site of contact and interaction between many microorganisms and the immune system, an association between salivary microbiota and CSU has not yet been reported. Thus, this study aimed to identify and compare differences in salivary microbiota composition between patients with CSU and healthy subjects.

## 2. Results

### 2.1. Demographic and Clinical Characteristics of Participants

The study included 23 participants, 13 patients with CSU, and 10 healthy controls (HC) (Table 1). There were no significant differences in gender and age between the observed groups.

Clinical and laboratory parameters of CSU patients are provided in Table 2.

At the time of enrollment in the study, all patients were under treatment with 2nd-generation H1-antihistamines. Up-dosing antihistamines (up to 4 times the standard dose) was required in 85% of patients. None of the patients had been treated with oral corticosteroids, biologics, or immunosuppressants. Disease activity was assessed by UAS (Urticaria activity score) test (Table 3).

### 2.2. Composition of Salivary Microbiota in CSU Patients and HC

**Figure 1:**
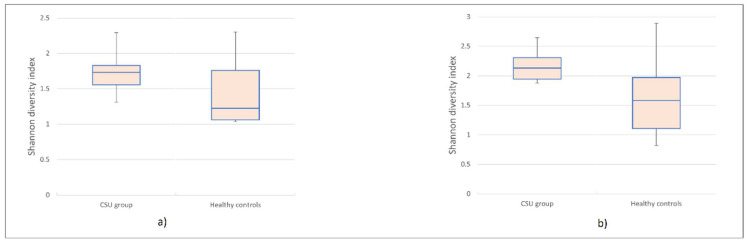
Boxplot was used to display the differences in alpha diversity visually. The Shannon index was significantly lower in Chronic Spontaneous Urticaria (CSU) group compared to the HC group. (**a**) *HhaI*-digestion. (**b**) *MspI*-digestion.

Terminal restriction fragment length polymorphism (T-RFLP) analysis identified 96 Operational Taxonomic Units (OTUs) in HhaI, and 102 OTUs in *MspI*-digested amplified 16S rRNA samples. More specifically, among 96 *HhaI*-digested OTUs, 79 OTUs were identified in the control group, and 69 OTUs were identified in the CSU group. From 102 OTUs digested by *MspI*, 91 OTUs were identified in the control group, whereas 77 OTUs were identified in the CSU group. Based on the results of *HhaI*/*MspI*-digested T-RF patterns, microbial diversity in CSU patients’ saliva samples was reduced compared to HC. Lower biodiversity was observed in datasets acquired from T-RFLP analysis of *HhaI* (*p* = 0.007, *t*-test) and *MspI* (*p* = 0.028, *t*-test) digested 16S rRNA fragments. The Shannon diversity index was significantly higher in the HC group compared to the CSU group (Figure 1a,b).

**Figure 2:**
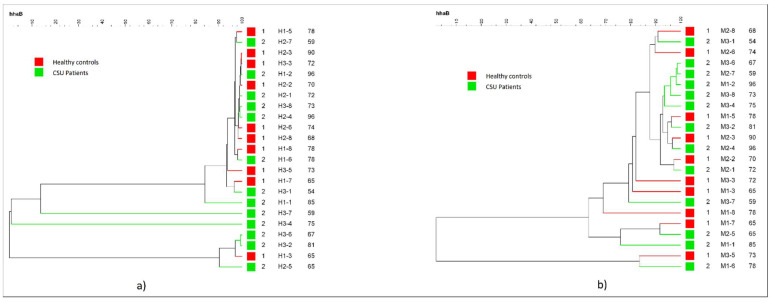
Dendogram of the salivary microbiota profiles of Chronic Spontaneous Urticaria (CSU) patients and HC. (**a**) *HhaI*-digestion. (**b**) *MspI*-digestion.

Dendrogram constructed by using a minimal variance algorithm did not reveal any major cluster characteristic for either group in neither *HhaI* nor *MspI* digested T-RF patterns (shown in Figure 2a,b). This study found no correlation between the severity of urticaria (evaluated by the UAS score) and microbial diversity. The post-hoc analysis did not identify any statistically significant difference in OTU abundance between the CSU and HC groups. The dominant phylum in the CSU group (according to the *HhaI* and *MspI*-digested T-RF patterns) was Proteobacteria, while Firmicutes were more abundant in the HC group. There was no significant difference at the phylum level in Firmicutes, Bacteroidetes, and Proteobacteria between the groups. OTUs representing the genus *Fretibacterium* were observed only in the control group. OTUs representing the genus *Pseudomonas* were more abundant in the CSU group. OTUs representing the genera *Bacteroides*, *Haemophilus*, *Bifidobacterium*, *Capnocytophaga*, *Fretibacterium*, *Porphyromonas* were more abundant in the HC group than in the CSU group.

## 3. Discussion

This case-control study aimed to identify and compare salivary microbial composition between CSU patients and HC. Our major findings revealed that alpha diversity of salivary microbiota in CSU patients was significantly reduced compared to HC. The Shannon index was significantly lower in the CSU group than in HC, which is highly consistent with other microbiome studies in CSU patients [6,11,22]. Beta diversity revealed that microbial composition was comparable between groups, without significant differences. Loss of microbial diversity leads to dysbiosis, which should be considered as an important underlying cause of immune-mediated diseases.

Due to the lack of previous studies in this field, we compared our results with recent reports of the connection between gut microbiota and CSU and the association between salivary microbiota and other immune-mediated diseases. There were a few overlaps between the salivary microbial composition in our CSU group and previously published reports about the implication of gut microbiota in CU/CSU. Based on our research, the dominant phylum in the CSU group was Proteobacteria, in contrast to the oral microbial composition of healthy individuals, where the most abundant microbes on the phylum level are Firmicutes [16]. However, increased Proteobacteria were observed in recent studies on gut dysbiosis in CU/CSU patients [10,14]. Increased Proteobacteria in the gut drastically enhance the permeability of the normally sterile mucus inner layer to the more penetrable region. That results in the infiltration of bacteria into the intestinal inner layer close to the epithelium [23]. Furthermore, Proteobacteria were increased in patients with asthma and allergic diseases; it was reported that Proteobacteria might cause allergies by upregulating Th17-related genes [24,25]. Increased Th17 cells were detected in CSU patients, suggesting that CSU development can be associated with Th17 cells. [26]. Our study showed a lower abundance of OTUs representing the genus *Bacteroides* in the CSU group, which is consistent with the study of Wang et al. [11] about the composition of the gut microbiome in CSU. Recently, it has been reported that unsaturated fatty acids, well-known for their anti-inflammatory properties [27,28], also have a stimulating effect on the growth of *Bacteroides* [29]. Wang et al. reported that the reduction in unsaturated fatty acids exacerbated inflammatory responses and triggered CSU development [11]. The abundance of *Bifidobacterium* was decreased in the CSU group compared to HC. The lower amount of *Bifidobacterium* was also reported in studies by Wang et al. on CSU patients [11], and Rezazadeh et al. on CU patients [30]. Some species of *Bifidobacterium* have anti-inflammatory effects; some strains are used as probiotics to improve dysbiosis and therefore alleviate inflammatory response [31]. A possible mechanism is by inducing regulatory T (Treg) cells [32,33]. Treg cells further induce the secretion of anti-inflammatory mediators [34], and therefore, can reduce inflammation. Reduced number and function of Treg in CU patients were shown in some studies [34,35]. OTUs representing the genus *Haemophilus* were more abundant in the control group than in the CSU group. For comparison, a study by Zhang et al. reported depletion of the *Haemophilus* species in saliva, dental plaque, and fecal samples in patients with rheumatoid arthritis, which was partly normalized after treatment of rheumatoid arthritis [18].

Gut and oral environments are infinitely complex, and the microbiota of these environments plays an important role in maintaining homeostasis. With reduced exposure of the gut microbiota to the immune system, a significant increase in the incidence and prevalence of allergic as well as autoimmune and inflammatory disorders has been reported. [36]. Although the current literature largely suggests gut dysbiosis as the most critical in the development of immune imbalance, there is a growing number of research suggesting the capability of oral bacteria to disrupt the gut microbial composition and induce chronic inflammation, and therefore trigger or exacerbate certain diseases [37,38,39].

Salivary bacteria are continuously swallowed, can spread and colonize other areas, and thus, may colonize, shape, and influence the gut microbiota [21]. An association between the oral and gut microbiota has been observed in rheumatoid arthritis, gastrointestinal cancer, and inflammatory bowel disease [18,39,40]. We speculate that there is a possible correlation between salivary and gut microbiota in CSU also. Therefore, there is an unmet need for studying microbiome at different sites along the digestive tract to better understand dysbiosis and its impact on the immune system.

Our study has several limitations. A small number of subjects are included, which probably resulted in nonsignificant statistical trends in the abundance of bacteria. We performed not so powerful molecular method which could detect bacteria at the species level; we only characterized the microbiota from one body site. Also, many clinical, demographic, and environmental factors (age, gender, dietary intake, lifestyle habits) can affect the salivary microbiota; so more extensive research is required to confirm whether microbial dysbiosis is associated exclusively with disease or other environmental factors are included. Although T-RFLP may not be as sufficient at the species-level classification of bacteria as next-generation sequencing (NGS), it is a reliable technique and a good tool for comparing microbial communities and assessing diversity in complex microbial communities [41].

To our knowledge, this was the first study that found changes in salivary microbiota in CSU patients. However, this study is in the exploratory stage. Larger and sufficiently powered studies are needed to analyze genomic, structural, and functional differences of salivary and gut microbiota and investigate the correlation between these microbial sites.

In conclusion, our study revealed changes in the composition of salivary microbiota in CSU patients. Dysbiosis of salivary microbiota may contribute to the dysregulated immune system in the development of CSU.

Further clinical studies on the salivary microbiome are needed to investigate the usefulness of salivary analysis for potential clinical and practical implications in CSU management.

## 4. Materials and Methods

### 4.1. Study Design and Sample Collection

Twenty-three participants (13 patients with CSU and 10 healthy controls) were enrolled in the study at the Department of Dermatology and Venereology, Sestre milosrdnice University Hospital Centre, Zagreb, Croatia. The study was approved by the Research Ethics Committee of Sestre milosrdnice’s University Hospital Centre, Zagreb (Approval No. EP-8247/19-10; 9 May 2019). Written informed consent was obtained from all the participants.

Inclusion criteria were: patients aged 18 or older with diagnosed CSU according to EAACI/GA LEN/EDF/WAO guidelines [1]. Exclusion criteria were: taking systemic antibiotics in the last three months, chronic smoking or alcohol consumption, periodontitis, history of rheumatoid arthritis, metabolic diseases (diabetes type I and II, obesity), gastrointestinal diseases (celiac disease, ulcerative colitis, Chron disease), malignant diseases and pregnancy. A dermatovenerologist examined all patients with CSU. The dynamic and severity of the disease were evaluated via the Urticaria Activity Score questionnaire (UAS) [1]. Saliva samples were collected from all 23 participants, who were asked not to drink or eat for at least 2 h before sampling. Saliva samples were collected according to a standard technique: 5 mL of spontaneous, whole, unstimulated saliva was collected from each participant into a 50 mL sterile DNA-free conical tube and stored at −80 °C. According to the manufacturer’s instructions, total bacterial DNA extraction from 250 uL of saliva samples was performed using the ZymoBIOMICS DNA Miniprep Kit (Zymo Research, Irvine, CA, USA).

### 4.2. PCR Amplification and T-RFLP Analysis

Polymerase chain reaction (PCR) amplification of a total 16S rDNA and T-RFLP analysis were performed according to Andoh et al. [42]. 6′-carboxyfluorescein (6-FAM) labeled 27-F primer (6-FAM-5′-AGAGTTTGATCCTGGCTCAG-3′), and 1492R (5′-GGTTACCTTGTTACGACTT-3′) (Thermo Fisher Scientific, Waltham, MA, USA) were used to amplify total 16S rDNA from the human saliva samples [42,43]. The PCR amplification of a total of 16S rDNA was performed in triplicates, in a total volume of 50 µL as described previously [44,45]. Purification of Amplified DNA was performed by QIAquick PCR Purification Kit (Qiagen, Hilden, Germany). 120 ng of purified PCR product was digested separately by *HhaI* and *MspI* enzymes (Thermo Fisher Scientific, Waltham, MA, USA) [42]. Restriction products were purified by ethanol/sodium acetate/EDTA precipitation and resuspended in deionized formamide to a final concentration of 10 ng/µL (Thermo Fisher Scientific, Waltham, MA, USA) [28]. T-RFLP analysis service (Macrogen Europe BV, Amsterdam, The Netherlands) on ABI PRISM 3730XLs (Thermo Fisher Scientific, Waltham, MA, USA), using 1200LIZ size standard, was used to obtain T-RFLP profiles of every sample. GeneMapper 3.7 software (Thermo Fisher Scientific, Waltham, MA, USA) was used for fragment size estimation. All T-RFs sized 50–900 bp with a peak height over 25 fluorescence units were included in further analysis. T-REX software (http://trex.biohpc.org/, accessed date November 2020) was used for the alignment of terminal restriction fragments (T-RFs) [46]. A binning threshold of 2 bp was used to assign T-RFs to OTUs [47].

The OTUs were quantified as the percentage values of individual OTU areas per total OTU area. This was expressed as the percentage of the area of the under peak curve (AUC) [48]. In silico assignment of OTUs to bacterial taxa listed in the Oral Micriome CORE Database (version 13 October 2017) was performed by PAT+ tool as a part of MiCA3 (http://mica.ibest.uidaho.edu/pat.php, accessed date November 2020) [49].

### 4.3. Statistical Analysis

Categorical data were analyzed by chi-square test. The age difference between groups was compared by using a student’s *t*-test. The Shannon-Wiener diversity index was calculated to compare diversity between CSU and HC groups, using the relative abundance of OTUs. The statistical difference between the indices was further calculated using the student’s t-test. Correlation between UAS score and Shannon-Wiener diversity index in the CSU group was assessed by calculating Pearson’s correlation coefficient. Dendrograms representing calculated similarity distances were generated using Pearson’s similarity coefficient analysis and the unweighted pair-group methods with arithmetic means (UPGMA). Dendrograms based on the *HhaI* or *MspI* T-RFLP patterns were generated by BioNumerics software (Applied Maths, Sint-Martens-Latem, Belgium). Statistical analysis was performed using SPSS 19.0 (Chicago, IL, USA), *p* values less than 0.05 were considered statistically significant.

## Figures and Tables

**Table 1:** Baseline characteristics of CSU patients and HC.

Variable	CSU Patients (*n* = 13)	Healthy Controls (*n* = 10)
Age (years), mean (SD)	46 (13)	47 (7)
Female, *n* (%)	11 (85%)	8 (80%)
Duration of symptoms (months), mean (SD)	9.2 (5.8)	-

**Table 2:** Clinical and laboratory parameters of CSU patients.

Variable	CSU Patients (*n* = 13)
Associated angioedema *n* (%)	7 (54%)
Associated atopic disorders, *n* (%)	4 (31%)
Associated thyroid disorders, *n* (%)	7 (54%)
Associated depression, *n* (%)	4 (31%)
Elevated TPO-Ab, *n* (%)	5 (38%)
Positive ANA, *n* (%)	6 (46%)
Elevated IgE	5 (38%)
Vitamin D levels Deficiency Insufficiency	5 (38%) 1 (8%)

Abbreviation: TPO-Ab, thyroid peroxidase antibodies; ANA, antinuclear antibodies.

**Table 3:** UAS score.

Variable	CSU Patients (*n* = 13)
UAS score	
Mild (7–15)	1 (8%)
Moderate (16–27)	6 (46%)
Severe (28–42)	6 (46%)

Abbreviation: UAS, urticaria activity score.

## Data Availability

The data presented in this study are available on request from the corresponding author. The data are not publicly available due to the personal data of the study participants.
